# Relevance of the time interval between surgery and adjuvant radio (chemo) therapy in HPV-negative and advanced head and neck carcinoma of unknown primary (CUP)

**DOI:** 10.1186/s12885-021-08885-3

**Published:** 2021-11-18

**Authors:** Matthias Balk, Robin Rupp, Konstantin Mantsopoulos, Moritz Allner, Philipp Grundtner, SK Mueller, Maximilian Traxdorf, Markus Eckstein, Stefan Speer, Sabine Semrau, Rainer Fietkau, Heinrich Iro, Markus Hecht, Antoniu-Oreste Gostian

**Affiliations:** 1 Department of Otolaryngology, Head & Neck Surgery, University Hospital Erlangen, Friedrich-Alexander University Erlangen-Nuremberg, 91054 Erlangen, Germany; 2 Department of Pathology, University Hospital Erlangen, Friedrich-Alexander University Erlangen-Nuremberg, Erlangen, Germany; 3 Department of Radiation Oncology, University Hospital Erlangen, Friedrich-Alexander University Erlangen-Nuremberg, Erlangen, Germany

**Keywords:** CUP-syndrome, Delayed adjuvant therapy, Advanced head and neck cancer, Neck dissection

## Abstract

**Introduction:**

In contrast to head and neck squamous cell carcinoma (HNSCC), the effect of treatment duration in HNSCC-CUP has not been thoroughly investigated. Thus, this study aimed to assess the impact of the time interval between surgery and adjuvant therapy on the oncologic outcome, in particular the 5-year overall survival rate (OS), in advanced stage, HPV-negative CUPs at a tertiary referral hospital. 5-year disease specific survival rate (DSS) and progression free survival rate (PFS) are defined as secondary objectives.

**Material and methods:**

Between January 1st, 2007, and March 31st, 2020 a total of 131 patients with CUP were treated. Out of these, 59 patients with a confirmed negative p16 analysis were referred to a so-called CUP-panendoscopy with simultaneous unilateral neck dissection followed by adjuvant therapy. The cut-off between tumor removal and delivery of adjuvant therapy was set at the median, i.e. patients receiving adjuvant therapy below or above the median time interval.

**Results:**

Depending on the median time interval of 55 days (d) (95% CI 51.42–84.52), 30 patients received adjuvant therapy within 55 d (mean 41.69 d, SD = 9.03) after surgery in contrast to 29 patients at least after 55 d (mean 73.21 d, SD = 19.16). All patients involved in the study were diagnosed in advanced tumor stages UICC III (*n* = 4; 6.8%), IVA (*n* = 27; 45.8%) and IVB (*n* = 28; 47.5%).

Every patient was treated with curative neck dissection. Adjuvant chemo (immune) radiation was performed in 55 patients (93.2%), 4 patients (6.8%) underwent adjuvant radiation only. The mean follow-up time was 43.6 months (SD = 36.7 months).

The 5-year OS rate for all patients involved was 71% (95% CI 0.55–0.86). For those patients receiving adjuvant therapy within 55 d (77, 95% CI 0.48–1.06) the OS rate was higher, yet not significantly different from those with delayed treatment (64, 95% CI 0.42–0.80; X^2^_(1)_ = 1.16, *p* = 0.281).

Regarding all patients, the 5-year DSS rate was 86% (95% CI 0.75–0.96). Patients submitted to adjuvant treatment in less than 55 d the DSS rate was 95% (95% CI 0.89–1.01) compared to patients submitted to adjuvant treatment equal or later than 55 d (76% (95% CI 0.57–0.95; X^2^_(1)_ = 2.32, *p* = 0.128). The 5-year PFS rate of the entire cohort was 72% (95% CI 0.59–0.85). In the group < 55 d the PFS rate was 78% (95% CI 0.63–0.94) and thus not significantly different from 65% (95% CI 0.45–0.85) of the group ≥55 d; (X^2^_(1)_ = 0.29, *p* = 0.589).

**Conclusions:**

The results presented suggest that the oncologic outcome of patients with advanced, HPV-negative CUP of the head and neck was not significantly affected by a prolonged period between surgery and adjuvant therapy. Nevertheless, oncologic outcome tends to be superior for early adjuvant therapy.

## Introduction

In about 2–5% of head and neck cancer patients with cervical lymph node metastases no primary tumor location can be found [1–3], leading to the diagnosis “cancer of unknown primary” (CUP).

Although various entities have been reported, the most common histology is squamous cell carcinoma accounting for 53–77% of the cases [4]. In this regard, the differentiation between human papilloma virus (HPV)-positive and -negative CUP-syndromes has been shown to influence treatment outcome with favorable outcomes for HPV-positive CUP [5]. However, HPV prevalence differs substantially from 22 and 91% between studies [2, 5–8]. Information on the 5-year survival rates varies impressively between 29 and 82% according to the relevant literature [8–12].

For early-stage disease treatment options include ipsilateral selective neck dissection followed by radiation therapy or chemoradiation or primary radiation / chemoradiation therapy that allow for comparable survival rates of up to 61.2% after 5 years [13].

For patients with a single positive cervical node without extranodal extension treated by a comprehensive neck dissection defined as dissection of levels II-IV including at least 18 identified lymph nodes adjuvant radiotherapy may be avoided if the compliance allows for a thorough surveillance of the patient [14]. In contrast, locally advanced disease, i.e. UICC tumor stage III and IV, require a combined approach consisting of neck dissection followed by adjuvant radiation / chemoradiation [15–19].

In accordance with the general recommendation for head and neck squamous cell carcinoma (HNSCC) by Chen et al., adjuvant therapy for CUP is also recommended to be implemented within 6 weeks following ablative surgery [20]. Apart from the necessity for adjuvant therapy for advanced stage disease, there exist no valid analyses on the time range between surgery and adjuvant therapy for CUPs.

This study focusses solely on HPV negative CUPs and assesses as primary objective the impact of the time range between surgery and adjuvant therapy on the oncologic outcome, in particular the 5-year overall survival rate, at a tertiary referral hospital. The recurrence of disease, i.e. regional metastases and distant metastases, the 5-year disease specific survival rate and the 5-year progression free survival rate were defined as secondary objectives.

## Patients and methods

This retrospective cohort study was conducted at a single tertiary referral and academic cancer center. It was carried out according to the Declaration of Helsinki and approved by the local Ethics Committee (approval number 428_20 Bc).

All patients diagnosed with a p16 negative CUP-syndrome between January 1st, 2007, and March 31st, 2020, were included. The following inclusion criteria were applied: surgical treatment at our institution, histologically confirmed squamous cell carcinoma, complete medical and surgical record available, confirmed negative p16-status. The association with HPV was confirmed by overexpression of the surrogate marker p16INK4a.

The following exclusion criteria were applied: histological types other than squamous cell carcinoma, detection of the primary cancer, positive or unknown p16-status or discontinued treatment.

Smoking was defined as current smokers with a smoking history of at least more than 10 pack years, Alcohol consumption was defined as reported daily alcohol intake.

The treatment process for CUPs involved firstly a “no-touch”-panendoscopy followed either by a core needle biopsy or node picking of the suspicious lymph node. Subsequently, a positron emission tomography (PET) was performed. If there was no evidence of the primary cancer, the PET was followed by a so-called CUP-panendoscopy that included a curettage of the nasopharynx, bilateral tonsillectomy and multiple biopsies of the tongue base. Simultaneously, the respective neck dissection was performed on the side of the previously diagnosed malignant lymph node. In case of contralateral suspicious lymph nodes a bilateral neck dissection was performed.

Based on the median time interval between upfront neck dissection and implementation of adjuvant therapy, the patients were divided into two groups resulting in one group with less than the median (early treatment group) and one group with above the median (delayed treatment group) until start of the adjuvant therapy.

Adjuvant treatment consisted of either radiation therapy or chemoradiation. Indications for chemoradiation were extranodal extension and more than one affected lymph node [14].

Neck dissection was classified according to Robbins et al. where selective neck dissection denotes preservation of one or more groups of lymph nodes, modified radical neck dissection denotes preservation of one or more non-lymphatic structures and radical neck dissection denotes removal of the spinal accessory nerve, the sternocleidomastoid muscle and the internal jugular vein besides the lymph node groups [21]. Furthermore the Lymph Node Ratio (LNR) was determined and was defined as the number of positive lymph nodes divided by the total number of lymph nodes removed [22, 23].

Radiation techniques included 3D conformal radiation therapy, intensity-modulated radiation therapy (IMRT), or volumetric modulated arc therapy (VMAT). The dosage of radiation was 64Gray (Gy) in the area of the affected lymph node and 56Gy in the ipsilateral neck. The contralateral neck and possible primary sites (i.e. tonsils bilaterally, tongue base and hypopharynx) received 50Gy. A dose of 70Gy was delivered to the nasopharynx. Treatment was delivered either in single doses of 2.0Gy sequentially in shrinking field technique or as simultaneous integrated boost using single doses of up to 2.3Gy up to biologically equivalent cumulative doses. The standard approach for concomitant chemotherapy was two cycles of 5-Fluorouracil (800 mg/m^2^ body surface area (BSA) continuous infusion d1–5) in combination with Cisplatin (100 mg/m^2^ BSA) or Carboplatin (AUC 5) splitted to 3–5 days.

### Study objectives

Recurrence of disease was defined as regional tumor recurrence or distant metastasis. The primary objective was the 5-year overall survival rate and was calculated from the date of the neck dissection to the date of death from any cause. The secondary objectives such as the 5-year disease specific survival rate was calculated from the date of the neck dissection to the date of death from the disease. Additionally, the 5-year progression free survival rate was calculated from the date of the neck dissection to the date of progression of the disease. Patients that were still alive at the time of the follow-up cut-off were censored. Follow-up consisted of a clinical examination with a thorough ultrasound performed by an ENT specialist covering both sides of the neck every 6 weeks in the first year after the disease, every three months in the second and third year and every six months in the fourth and fifth year after finishing the treatment of the disease. A computed tomography scan of the neck and thorax was performed once a year. The tumor stage was determined according to the 8th version of the UICC [24].

### Statistical analysis

Continuous variables were tested for normal distribution using the Kolmogoroff-Smirnov test and each variable’s histogram. Consequently, normally distributed parameters are presented as mean ± 1 standard deviation (SD) and compared via independent T-Tests. In case of non-normally distributed variables, the median [1.; 3. Quartile] is additionally presented, and group comparisons were performed with the Mann-Whitney-*U*-test.

Categorical variables are presented as absolute and relative values (N / %) and compared with the Chi-Square-Test or with the Exact Fischer-Test, whatever applicable. Survival rates were created by using the Kaplan-Meier-Method and compared by the Log-Rank-Test. Overall-, disease-specific and progression free survival values are presented as Kaplan-Meier estimates and 95% confidential intervals (CI). Effect sizes for chi^2^-tests are reported as phi in case of nominal variables with 2 levels. A phi of 0.1 displays a small effect, 0.3 a medium, and 0.5 a strong effect [25].

A *p*-value of less than 0.05 was considered statistically significant. Statistical analysis was performed using IBM SPSS Statistics, version 26.0 (IBM Corp., Armonk, NY, USA).

## Results

### Patient characteristics

Between January 1st, 2007, and March 31st, 2020 a total of 131 patients were treated with a CUP-syndrome at our Department. Out of these, 59 patients with a confirmed negative p16 status were referred to a so-called CUP-panendoscopy with simultaneous neck dissection followed by adjuvant therapy. The entire patient cohort averaged 60.76 years (yrs) (SD = 10.42 yrs) and included 49 male patients (83.1%).

The neck dissection was performed in 23 patients on the left side (39%), in 32 patients on the right side (54.2%) and in 4 patients simultaneously on both sides (6.8%).

Both groups did not differ significantly with respect to gender (X^2^_(1)_ = 0.57, *p* = 0.451), age (t_(57)_ = 1.97, *p* = 0.054), nodal stage (X^2^_(4)_ = 2.99, *p* = 0.559), UICC stage (X^2^_(2)_ = 1.16, p = 0.559, there was no stage I or II), extranodal extension (X^2^_(1)_ = 0.61, *p* = 0.436), noxious agents (smoking X^2^_(1)_ = 0.14, *p* = 0.711, alcohol X^2^_(1)_ = 3.78, *p* = 0.151) and the ASA-Score (X^2^_(2)_ 0.35, *p* = 0.841). The most common reasons for delayed initiation of adjuvant therapy were organizational reasons, patient indecision, and in few cases delayed wound healing.

All patients were diagnosed in advanced tumor stages UICC III (*n* = 4; 6.8%), IVA (*n* = 15; 25.4%) and IVB (*n* = 40; 67.8%) with complete absence of early tumor stages I and II (Table 2). Furthermore, 40 out of 59 patients (67.8%) had a pN3b nodal stage. Patients’ characteristics are presented in Table 2 revealing no significant differences between both patient groups.

### Treatment characteristics

All patients were treated with curative neck dissection of whom 33 patients (55.9%) received a selective neck dissection, 10 patients (17%) a modified radical neck dissection and 16 patients (27.1%) underwent a radical neck dissection. Adjuvant chemo (immune) radiation was performed in 55 patients (93.2%) whereas 4 patients (6.8%) underwent adjuvant radiation only. The mean follow-up time was 43.59 months (SD = 36.71 months). The median duration from curative tumor removal to the beginning of adjuvant therapy was 55 d (95% CI 51.42–84.52).

Accordingly, 30 patients received adjuvant therapy within less than 55 d after surgery in contrast to 29 patients that received adjuvant therapy after at least 55 d.

The early treatment group started with adjuvant therapy following ablative surgery on average after 41.69 d (SD = 9.03) in contrast to the delayed adjuvant treatment starting on average after 73.21 d (SD = 19.16; t_(56)_ = 8.01, *p* < 0.001).

Treatment modalities were similarly distributed between both treatment groups with respect to the surgical treatment modality (selective neck dissection, modified radical neck dissection, radical neck dissection; X^2^_(2)_ = 1.74, *p* = 0.419), the number of removed lymph nodes (t_(57)_ = 0.47, *p* = 0.639), regarding the Lymph Node Ratio (Z = 1.11, *p* = 0.268), the adjuvant treatment modality (radiation therapy, chemoradiation therapy; Fisher’s Z: *p* > 0.999), the radiation dose in Gray (Z = 0.49, *p* = 0.621) and the chemotherapy regimen (X^2^_(1)_ = 0.44, *p* = 0.508; Tables 1, 2 and 3). One patient refused chemotherapy and was treated with radiation therapy only. Tables 1, 2 and 3 display the patient´ characteristics and applied treatment modalities.

**Table 1:** Patients´ characteristics

	All patients (*n* = 59)	Early treatment group (*n* = 30)	Delayed treatment group (*n* = 29)	Statistical comparison
Gender (n, %)				
Male	49 (83.0%)	26 (86.7%)	23 (79.3%)	X^2^_(1)_ = 0.57, *p* = 0.451
Female	10 (17.0%)	4 (13.3%)	6 (20.7%)	
Age (mean years ± SD)	60.76 ± 10.42	58.43 ± 9.39	63.17 ± 11.03	t_(57)_ = 1.97, *p* = 0.054
Nodal stage (n, %)				
pN1	5 (8.5%)	4 (13.3%)	1 (3.5%)	
pN2a	5 (8.5%)	3 (10.0%)	2 (6.9%)	
pN2b	9 (15.2%)	4 (13.3%)	5 (17.2%)	X^2^_(3)_ = 2.20, *p* = 0.533
pN2c	0 (0%)	0 (0%)	0 (0%)	
pN3a	0 (0%)	0 (0%)	0 (0%)	
pN3b	40 (67.8%)	19 (63.4%)	21 (72.4%)	
UICC stage (n, %)				
III	4 (6.8%)	3 (10.0%)	1 (3.5%)	X^2^_(2)_ = 1.15, *p* = 0.563
IVA	15 (25.4%)	8 (26.7%)	7 (24.1%)	
IVB	40 (67.8%)	19 (63.3%)	21 (72.4%)	
Extranodal extension (n, %)				
Yes	42 (71,19%)	20 (66.7%)	22 (75.9%)	X^2^_(1)_ = 0.61, *p* = 0.436
No	17 (28.81%)	10 (33.3%)	7 (24.1%)	
Noxious agents				
Smoking	42 (71.2%)	22 (73.3%)	20 (69.0%)	X^2^_(1)_ = 0.14, *p* = 0.711
Alcohol	41 (69.5%)	19 (63.3%)	22 (75.9%)	X^2^_(1)_ = 3.78, *p* = 0.151
ASA-Score				
1	5 (8.5%)	3 (10.0%)	2 (6.9%)	X^2^_(2)_ 0.35, *p* = 0.841
2	47 (79.7%)	23 (76.7%)	24 (82.7%)	
3	7 (11.8%)	4 (13.3%)	3 (10.3%)	

Abbreviations: Early treatment group = adjuvant treatment was implemented in less than 55 d after surgery; Delayed treatment group = adjuvant treatment was implemented within or later than 55 d following surgery; *SD* standard deviation; *UICC* International Union Against Cancer; *ASA* American Society of Anesthesiologists

**Table 2:** Treatment characteristics

	All patients (*n* = 59)	Early treatment group (*n* = 30)	Delayed treatment group (*n* = 29)	Statistical comparison
Time span				
Neck Dissection – Adjuvant Therapy (mean d ± SD)	57.45 ± 21.75	41.69 ± 9.03	73.21 ± 19.16	t_(56)_ = 8.01, *p* < 0.001
Surgical treatment modality (n, %)				
Selective Neck Dissection	33 (55.9%)	19 (63.3%)	14 (48.3%)	
Modified Radical Neck Dissection	10 (17.0%)	5 (16.7%)	5 (17.2%)	X^2^_(2)_ = 1.74, *p* = 0.419
Radical Neck Dissection	16 (27.12%)	6 (20.0%)	10 (34.5%)	
Adjuvant treatment modality (n, %)				
Radiation therapy	4 (6.8%)	2 (6.7%)	2 (6.9%)	Fisher’s Z: *p* > 0.999
Chemoradiation therapy	55 (93.2%)	28 (93.3%)	27 (93.1%)	
Radiation dose in Gy (mean ± SD)	69.84 ± 8.19	70.41 ± 8.88	69.25 ± 7.54	Z = 0.49, *p* = 0.621
Chemotherapy (n, %)				
Cisplatin/ Carboplatin+ 5-FU	31 (56.4%)	17 (60.7%)	14 (51.8%)	X^2^_(1)_ = 0.44, *p* = 0.508
Other	24 (43.6%)	11 (39.3%)	13 (48.2%)	
Number of removed lymph nodes	21.95 ± 10.70	21.30 ± 10.96	22.62 ± 10.61	t_(57)_ = 0.47, *p* = 0.639
Lymph Node Ratio (LNR; mean ± SD)	0.25 ± 0.43	0.31 ± 0.57	0.18 ± 0.21	Z = 1.11, *p* = 0.268

Abbreviations: Early treatment group = adjuvant treatment was implemented in less than 55 d after surgery; Delayed treatment group = adjuvant treatment was implemented within or later than 55 d following surgery; *SD* standard deviation; *Gy* Gray; *5-FU* 5-Fluorouracil

**Table 3:** Oncological outcomes

	All patients (*n* = 59)	Early treatment group (*n* = 30)	Delayed treatment group (*n* = 29)	Statistical comparison
Recurrence of disease:				
Local & Regional metastases	5 (8.5%)	3 (10.0%)	2 (6.9%)	X^2^_(1)_ = 1.57, *p* = 0.692
Distant metastases	11 (18.6%)	5 (16.7%)	6 (20.7%)	
5-year OS rate				
Events (death)	11 (18.6%)	4 (16.7%)	7 (24.1%)	X^2^_(1)_ = 1.16, *p* = 0.281*
KM Estimate (95%-CI)	0.71 [0.55;0.86]	0.77 [0.48;1.06]	0.64 [0.42;0.80]	
5-year DSS rate				
Events (death)	6 (10.2%)	1 (3.3%)	5 (17.2%)	X^2^_(1)_ = 2.32, *p* = 0.128*
KM Estimate (95%-CI)	0.86 [0.75;0.96]	0.95 [0.89;1.01]	0.76 [0.57;0.95]	
5-year PFS rate				
Events (death)	14 (23.7%)	6 (20.0%)	8 (27.6%)	X^2^_(1)_ = 0.29, *p* = 0.589*
KM Estimate (95%-CI)	0.72 [0.59;0.85]	0.78 [0.63;0.94]	0.65 [0.45;0.85]	

Abbreviations: Early treatment group = adjuvant treatment was implemented in less than 55 d after surgery; Delayed treatment group = adjuvant treatment was implemented within or later than 55 d following surgery; *OS* overall survival; *DSS* disease-specific survival; *PFS* progression-free survival; *KM* Kaplan-Meier. *Calculated with the Log-Rank-Test

### Oncologic outcomes

**Fig. 1:**
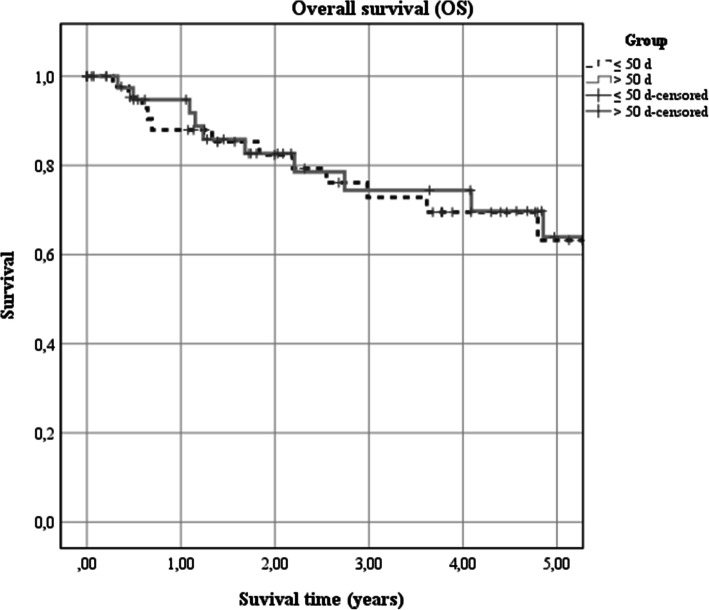
Association of time span from surgery to adjuvant therapy with overall survival. Kaplan-Meier estimates of overall survival according to the implementation of adjuvant therapy within (< 55 d) and later than 55 d (≥ 50 d) after surgery (X^2^_(1)_ = 1.16, p = 0.281)

Regarding all patients, the 5-year overall survival rate was 71% (95% CI 0.55–0.86). Patients receiving adjuvant therapy within 55 d (77, 95% CI 0.48–1.06) had a higher, yet not significantly different overall survival rate compared to those with delayed treatment (64, 95% CI 0.42–0.80; X^2^_(1)_ = 1.16, *p* = 0.281; Fig. 1).

**Fig. 2:**
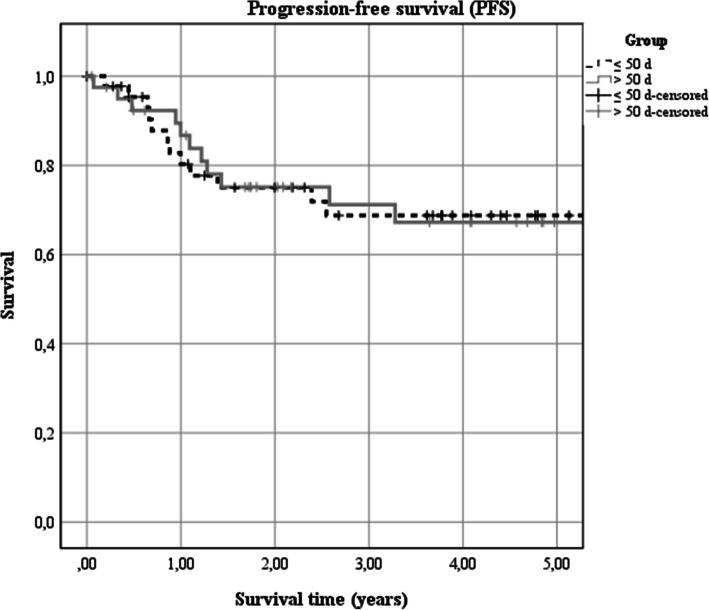
Association of time span from surgery to adjuvant therapy with progression free survival. Kaplan-Meier estimates of overall survival according to the implementation of adjuvant therapy within (< 55 d) and later than 55 d (≥ 55 d) after surgery (X^2^_(1)_ = 2.32, *p* = 0.128)

The 5-year disease-specific survival rate of all patients was 86% (95% CI 0.75–0.96). For patients receiving adjuvant treatment in less than 55 d it was 95% (95% CI 0.89–1.01) compared to patients receiving adjuvant treatment equal or later than 55 d (76% (95% CI 0.57–0.95; X^2^_(1)_ = 2.32, *p* = 0.128; Fig. 2).

**Fig. 3:**
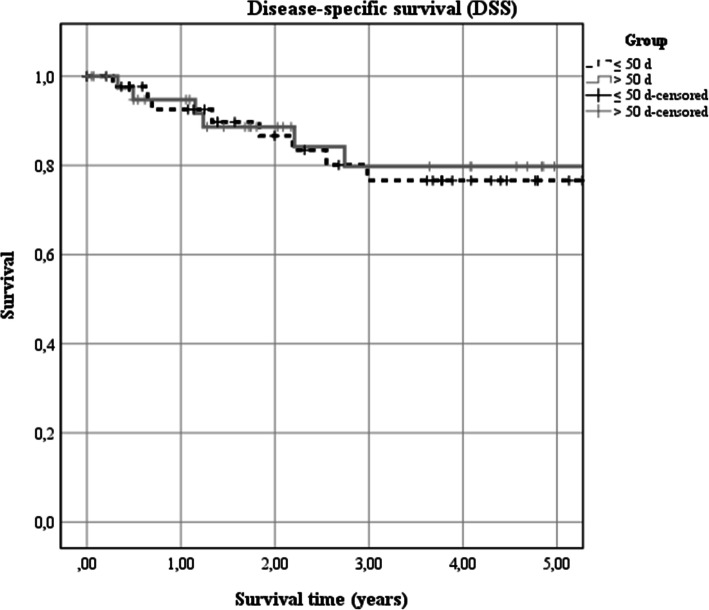
Association of time span from surgery to adjuvant therapy with disease specific survival. Kaplan-Meier estimates of overall survival according to the implementation of adjuvant therapy within (< 55 d) and later than 55 d (≥ 50 d) after surgery (X^2^_(1)_ = 0.29, *p* = 0.589)

The 5-year progression-free survival rate of the entire cohort was 72% (95% CI 0.59–0.85). In the early treatment group, it was 78% (95% CI 0.63–0.94) and thus not significantly different from 65% (95% CI 0.45–0.85) of the delayed treatment group (X^2^_(1)_ = 0.29, *p* = 0.589; Fig. 3).

In total, 5 patients (8.5%) experienced a regional recurrence, i.e. on the ipsilateral neck side, averaging 36.6 months (SD = 53.78) after adjuvant chemoradiation. Three patients (10%) of the early treatment group developed a regional recurrence averaging 51.33 months (SD = 68.99) after adjuvant therapy. Similarly, 2 out of 30 patients (6.9%) of the delayed treatment group developed a regional recurrence after 14.5 months (SD = 20.51) (Fisher’s Z: *p* > 0.999, phi = − 0.056).

Out of all surveyed patients, 11 patients (18.6%) presented with distant metastasis averaging 12.0 months (SD = 9.22) during follow up. Five out of 30 patients (16.7%) of the early treatment group developed distant metastasis averaging 9.2 months (SD = 4.97) after adjuvant therapy and 6 out of 29 patients (20.7%) of the delayed treatment group developed distant metastasis averaging 14.33 months (SD = 11.66) after adjuvant therapy (X^2^_(1)_ = 1.57, *p* = 0.692, phi = 0.052).

Patients with extranodal extensions were significantly more likely to have distant metastases compared with those who did not have extranodal extensions (11/42 (26.2%) vs. 0/17 (0%), Fisher’s z: p = 0,024, phi = 0,305). There was no difference regarding regional recurrence (ENE+: 4/42 (9.5%) vs. ENE-:1/17 (5,9%), Fisher’s z: *p* > 0.999, phi = 0,059).

## Discussion

The results of this study indicate that a prolonged time between surgery and the implementation of subsequent adjuvant therapy for advanced staged head and neck CUP at the cut-off of 55 days did not significantly compromise the 5-year overall survival rate, the 5-year disease-specific survival rate and the 5-year progression-free survival rate. However, early treatment tended to be superior. Additionally, the rate for regional recurrence and distant metastases was not affected distinctly by the time between surgical tumor removal and initiation of adjuvant therapy. Thus, adjuvant therapy should not be omitted, also in case of a delay.

In general, adjuvant therapy is recommended to be applied within a short time frame following surgery to avoid the growth of residual malignant cells and to maximize the effect of radiation therapy [26–28]. For head and neck cancer, Chen et al. recommend the implementation of adjuvant therapy within six weeks after surgery, although head and neck cancer of unknown primary was not explicitly reviewed [20]. In contrast to our results Grau et al. demonstrated in a Danish multicenter study that a prolonged overall treatment, i.e. at the cut-off of 50 d, significantly impaired neck control and regional tumor control but not survival. However, their analysis did not distinguish between HPV positive and negative CUPs and also included 20% cases with the appearance of a primary tumor during the study period [1, 29]. Apart from that there is no data regarding the oncologic outcome for prolonged adjuvant radiation therapy/ chemoradiation therapy after primary surgery in CUP-patients.

However, the effect of time to radiation therapy after surgery has already been studied for head and neck cancer in general. In their cohort study including 25.216 patients, Harris et al. reported that a rather shorter interval from the date of surgery to the start of radiation therapy within 42 days was associated with an improved survival for head and neck squamous cell carcinoma. The early treatment group in the presented study started with adjuvant therapy following surgery on average after 41.69 d. Interestingly, treatments that were carried out entirely at an academic center were associated with longer delays to start radiation (> 42 days) but still improved survival regardless of the HPV-status (HR 1.06; 95% CI, 1.01–1.12). This is in line with our observation for advanced staged head and neck CUP-Syndromes treated at our academic center, that delayed adjuvant therapy did not significantly compromise the overall survival rate [30].

The oncological outcome in general for the patients in this study with a 5-year overall survival rate of 71%, a 5-year disease-specific survival rate of 86% and a 5-year progression-free survival rate of 72% is comparable or even superior to available literature and demonstrates the improvement of the overall survival in the treatment of advanced-stage CUPs. The aforementioned study by Grau et al. which reported a 5-year overall survival rate of 37% resulting from unimodal treatment with radiation therapy during the period of 1975 to 1995 [1] while more recent studies investigating multimodal treatment report 5-year overall survival rates ranging from 40,9% to 78,9% [31–35]. Especially the low rate of regional recurrences of only 8.5% highlights the high efficacy of surgery followed by chemoradiation therapy. However, 18.6% of patients developed distant metastases, which indicates the need for improved systemic therapy. A possible future direction might be the integration of immunotherapy, which is efficient in recurrent/metastatic HNSCC [36, 37] and probably also in addition to induction chemotherapy for HNSCC [38] but seems to require adequate patient selection when administered concomitant to chemoradiation therapy of HNSCC [39].

The foremost limitation of this study is due to the inevitable bias of the retrospective character of the analysis. However, the study is based on a homogeneous cohort of patients with well comparable groups revealing similar characteristics. Nevertheless, the number of patients considered is comparable to available literature on this topic [6–8, 40]. Regarding the included patients, our results refer to advanced-stage head and neck CUPs that are HPV-negative and may not be readily generalizable to early-stage disease and/or HPV-positive CUPs. In addition, it must be mentioned that the administration of 70 Gy to the nasopharynx and 50 Gy to the contralateral tonsil, tongue base and hypopharynx is the traditionally established treatment regimen in our center. Since no relevant nasopharyngeal- or contralateral oro-/hypopharyngeal mucosal associated toxicities have occurred in recent decades, this treatment regimen was not changed during the period of the current analysis (2007-2020).

Of note, this study did not intend to define a definitive threshold time between surgery and the implementation of adjuvant therapy as this was beyond the scope of this study. This crucial topic warrants further investigations preferably multicenter to increase the number of included patients.

The presented results indicate that in head and neck-CUP patients a time span of more than 55 days from surgery to the implementation of adjuvant therapy does not significantly affect the 5-year overall survival rate, although there may be a benefit for those patients which received adjuvant therapy within 55 d. In conclusion, patients with advanced staged CUP-Syndrome may also benefit from delayed adjuvant therapy and therefore it should not be withheld from patients with a prolonged time in between surgery and adjuvant therapy.

## Conclusion

The results of this study demonstrate that delayed adjuvant therapy in head and neck-CUP-syndrome more than 55 days after surgery did not significantly impair the 5-year overall survival rate, the 5-year disease-specific survival rate and the 5-year progression-free survival rate. Nevertheless, oncologic outcome tends to be superior for early adjuvant therapy. Further studies with larger patient cohorts are needed to evaluate a definitive threshold time frame.

## Data Availability

The datasets used and/or analysed during the current study are available from the corresponding author on reasonable request.
